# Information booklet for promoting self-efficacy in childhood asthma: construction and validity

**DOI:** 10.1590/1980-220X-REEUSP-2022-0461en

**Published:** 2023-07-10

**Authors:** Flávia Ximenes Vasconcelos, Lorena Pinheiro Barbosa, Francisca Elisângela Teixeira Lima, Leidiane Minervina Moraes de Sabino, Kamila Ferreira Lima, Elizamar Regina da Rocha Mendes

**Affiliations:** 1 Universidade Federal do Ceará, Faculdade de Farmácia, Odontologia e Enfermagem, Departamento de Enfermagem, Fortaleza, CE, Brazil.; 2 Universidade da Integração Internacional da Lusofonia Afro-Brasileira, Departamento de Enfermagem, Fortaleza, CE, Brazil.

**Keywords:** Asthma, Child Health, Self Efficacy, Educational Technology, Validation Studies, Asma, Salud Infantil, Autoeficacia, Tecnología Educacional, Estudios de Validación, Asma, Saúde da Criança, Autoeficácia, Tecnologia Educacional, Estudos de Validação

## Abstract

**Objective:**

To construct and validate the content and appearance of an information booklet to promote self-efficacy of parents and/or caregivers in childhood asthma management and control.

**Method:**

This is a methodological study, developed from educational material elaboration, validity and assessment by 25 content judges and three technical judges. Language clarity, practical pertinence and theoretical relevance were used, calculating the Content Validity Coefficient (CVC) for validity, and the Suitability Assessment of Materials (SAM) instrument was applied for assessment. Judges were also able to make suggestions for modifications on each page of the booklet. Pages that reached a CVC ≥ 0.80 for content judges and CVC ≥ 0.70 for technical judges were considered validated.

**Results:**

The total CVC of the booklet was 0.96 for content judges and 0.83 for technical judges. The educational material was considered superior according to the SAM, with a total score of 92.67% for content judges and 73.81% for technical judges. Changes were made to the booklet after the validity process, according to judges’ suggestions, resulting in a second version.

**Conclusion:**

The information booklet is valid and has a high degree of recommendation for use with parents and/or caregivers in childhood asthma control and management.

## RESUMO

**Objetivo:**

Construir e validar conteúdo e aparência de álbum seriado para promoção da autoeficácia de pais e/ou cuidadores no manejo e controle da asma infantil.

**Método:**

Estudo metodológico, desenvolvido a partir da elaboração, validação e avaliação do material educativo por 25 juízes de conteúdo e três juízes técnicos. Utilizou-se clareza da linguagem, pertinência prática e relevância teórica, calculando-se Coeficiente de Validade de Conteúdo (CVC) para validação, e aplicou-se o instrumento *Suitability Assesment of Materials* (SAM) para avaliação. Os juízes puderam, ainda, fazer sugestões de modificações em cada página do álbum. Foram consideradas validadas as páginas que atingiram CVC ≥ 0,80 para juízes de conteúdo e CVC ≥ 0,70 para juízes técnicos.

**Resultados:**

O CVC total do álbum foi de 0,96 para juízes de conteúdo e 0,83 para juízes técnicos. O material educativo foi considerado superior segundo o SAM, com escore total de 92,67% para juízes de conteúdo e 73,81% para juízes técnicos. Alterações foram feitas no álbum após processo de validação, de acordo com as sugestões dos juízes, obtendo-se uma segunda versão.

**Conclusão:**

O álbum seriado é valido e possui alto grau de recomendação para utilização com pais e/ou cuidadores no controle e manejo da asma infantil.

## RESUMEN

**Objetivo:**

Construir y validar el contenido y la apariencia de un rotafolio para promover la autoeficacia de los padres y/o cuidadores en el manejo y control del asma infantil.

**Método:**

Estudio metodológico, desarrollado a partir de la elaboración, validación y evaluación del material educativo por 25 jueces de contenido y tres jueces técnicos. Se utilizó claridad de lenguaje, pertinencia práctica y pertinencia teórica, calculándose el Coeficiente de Validez de Contenido (CVC) para la validación, y se aplicó el instrumento Suitability Assesment of Materials (SAM) para la evaluación. Los jueces también pudieron sugerir modificaciones a cada página del álbum. Se consideraron validadas las páginas que alcanzaron un CVC ≥ 0,80 para jueces de contenido y CVC ≥ 0,70 para jueces técnicos.

**Resultados:**

El CVC total del álbum fue de 0,96 para los jueces de contenido y de 0,83 para los jueces técnicos. El material educativo fue considerado superior según la SAM, con una puntuación total de 92,67% para los jueces de contenido y 73,81% para los jueces técnicos. Se realizaron cambios en el álbum después del proceso de validación, de acuerdo con las sugerencias de los jueces, lo que resultó en una segunda versión.

**Conclusión:**

El rotafolio es válido y tiene un alto grado de recomendación para su uso con padres y/o cuidadores en el control y manejo del asma infantil.

## INTRODUCTION

Asthma is a chronic inflammatory disease characterized by respiratory symptoms, including dyspnea, wheezing, chest pain and cough, with variable obstruction of expiratory airflow^(1)^. Globally, about 339 million people have asthma, of which 60% are children^(2)^. In Brazil, it is estimated that there are 20 million people with asthma^(3)^. According to the Brazilian Health System Department of Informatics (DATASUS – *Departamento de Informática do Sistema Único de Saúde*) (www.datasus.saude.gov.br), in 2019, there were 79,433 hospitalizations for asthma in the country, predominating the age group from zero to 14 years (66.9%), and the number of deaths was 442, in the same year. DATASUS also shows that, from January to August 2022, there were 54,264 hospitalizations, of which 41.249 were of people up to 14 years old, and 352 deaths.

Hospitalizations and deaths due to asthma result from the inability to control the disease. To achieve asthma control and management, actions are needed to minimize exacerbations, such as continuous outpatient follow-up, prophylactic treatment, preventive measures in the home environment, education and family support, correct inhalation technique, treatment compliance and a written action plan^(1)^.

The knowledge and attitude of parents and/or caregivers of asthmatic children, related to disease symptoms, directly influence childhood asthma management. The prevalence of asthma is higher in children whose parents and/or caregivers lack knowledge. Thus, parents and caregivers who receive health education have more knowledge and better health behavior in relation to asthma, resulting in better controlling the disease in children^(4)^. In addition to knowledge, the importance of self-efficacy is highlighted, which is a person’s belief in their ability to organize and perform a certain task or behavior successfully^(5)^.

Recent research^(6,7)^ reveals that building parental confidence in providing care related to asthma influences and improves the quality of life of children and caregivers, which makes the theoretical construct of self-efficacy a basic component of educational interventions and essential for healthy behavior promotion. Therefore, in the teaching process and in users’ role, health professionals can use health technologies based on self-efficacy promotion of parents and caregivers in childhood asthma management and control, since research on asthma falls within the priorities of research in Brazil, according to the Brazilian National Agenda of Health Research Priorities (ANPPS – *Agenda Nacional de Prioridades da Pesquisa em Saúde*)^(8)^.

Studies that work on the development of self-efficacy through educational technologies related to asthma are still scarce. At the international level, educational interventions aimed at the self-efficacy of caregivers and children with asthma have obtained positive results using multimedia software^(9)^, videos and booklets^(10)^. At the national level, a booklet was recently developed entitled “You are able to control your child’s asthma. Shall we learn together?” (*Você é capaz de controlar a asma da sua criança. Vamos aprender juntos*?)^(11)^, whose results on effectiveness are yet to be released.

Among the health technologies currently available for educational interventions, printed technologies stand out here, which influence the communication process, in order to increase decision-making power and treatment compliance, through the provision of information that raises awareness of individuals^(11)^. Specifically, the information booklet constitutes a visual resource with a message in a logical and progressive way, sharing knowledge through texts and images and experiences and patient health situations through dialogue^(12,13)^.

Given the above, this study seeks to provide educational technology that allows for a dialogical relationship between health professionals and caregivers of children with asthma, to promote self-efficacy and contribute to health protection and promotion, consequently reducing child morbidity and mortality and hospitalizations due to asthma, generating less expenditure and improving the quality of health of children and families. Thus, the objective was to construct and validate an information booklet content and appearance to promote the self-efficacy of parents and/or caregivers in childhood asthma management and control.

## METHOD

### Study Design

This is a methodological study, in which an information booklet was developed to promote the self-efficacy of parents and/or caregivers in childhood asthma management and control, based on the elaboration, validity and assessment of the aforementioned educational material.

The booklet content was prepared based on the items of the Self-Efficacy and Their Child’s Level of Asthma Control scale- Brazilian version^(14)^ and on the educational booklet “Are you able to control your child’s asthma – shall we learn together?”^(11)^. It is noteworthy that the scale, the booklet and the aforementioned booklet were prepared in the light of Bandura’s Self-Efficacy Theory^(5)^. In addition, national guidelines from the Brazilian Society of Pneumology and Tisology^(3)^ and international guidelines from the Global Initiative For Asthma were considered^(1)^.

In this stage of booklet construction, two theoretical- methodological references were used that highlight important elements in the preparation of printed educational materials, in the development of illustrations and diagramming that would contribute to a better understanding of readers: Teaching patients with low literacy skill and Simply Put^(15,16)^.

To create the illustrations, an image bank was organized through a search on Google Images, using the following keywords: “airways”; “asthma”; “child”; “inhaler”; and “spacer”. Then, information booklet’s the textual script was elaborated and, later, a PowerPoint prototype was presented to a professional designer, who produced the illustrations and performed the diagramming of the first version of the educational material, using Adobe Illustrator CS3 and Photoshop CS6 programs.

The booklet took about eight months to build, from designing its initial model to illustration, diagramming and corrections.

### Site

The research was carried out linked to the *Universidade Federal do Ceará*. The stages for building the information booklet were developed in the city of Fortaleza, CE, Brazil. However, the data collection stages with content and technical judges and took place online, with participants from several states in the country.

### Population and Selection Criteria

Information booklet validation was carried out with content and technical judges, with the aim of giving greater credibility to the material prepared. A total of 25 content judges with teaching and care experience participated in the validity stage, being specialists in the field of child health; and three technical judges, being professionals in the field of communication and graphic design. The number of judges followed the recommendations suggested by Fehring and Lynn regarding the number of evaluators for this type of study^(17,18)^.

The sample selection was made for convenience. Thus, judges were selected, after searching the *Plataforma Lattes* for professionals, throughout Brazil, with a profile whose production and area of knowledge were compatible with the concept of this study. To this end, the keywords “asthma” and “child health” were used in the subject search mode, and the filter “professional practice” and “health sciences” was used to search for content judges^(11)^. Snowball sampling, as indicated by other professionals and research participants, was used in the search for technical judges and, in a complementary way, in the search for content judges.

After searching for professionals, the content and technical judges were then chosen based on Jasper’s criteria^(19)^, adapted with specific characteristics for each type of judge and related to the theme of asthma and educational materials. For content judges, Jasper’s criteria included education in the health field and experience in topics related to asthma, child health, family/collective/public health and respiratory diseases. For technical judges, criteria included involvement with educational materials, printed materials and information booklet.

Initially, 75 content judges were contacted. Of these, 50 did not respond to the email; 21 agreed to participate and assessed the material; three refused; and one agreed to participate but did not meet the deadline. For this reason, five new judges, indicated by other participants, were contacted. Only four agreed to participate and assessed the material in a timely manner. For technical judges, nine professionals were contacted. However, only one responded to the email and agreed to participate. This judge indicated two more possible participants, who agreed to be part of the research.

It is important to mention that content and technical judges were contacted at different times. Some of the judges responded to emails promptly. Regarding the contacted judges who did not receive a response after the first weeks of collection, a new email was sent, in order to maximize the chances of a response. Thus, the 15-day period only started after judges had agreed to participate, and it proved to be enough time for most professionals. However, deadline extension was necessary for some participants.

### Data Collection

Data collection for validity with judges took place from June to December 2020, from the selection of experts to the completion of their participation with response to the online instruments. After selecting the judges, all documents and collection instruments were sent via e-mail (invitation letter, Informed Consent Form, synthesis of Bandura’s Social Cognitive Theory, characterization questionnaire of judges, booklet validity instrument, Suitability Assessment of Materials instrument and digital copy of the booklet), hosted on an online platform (Google Forms) and provided through a link for direct access. After accepting participation, judges had a period of 15 days to finalize the booklet assessment.

The instrument used for validity was a Likert-type scale, with five points, ranging from “very little” to “very much”, which allows assessing each page of the booklet in terms of clarity of language, practical pertinence and theoretical relevance of Pasquali^(20)^. In addition to this, there was a space for judges’ suggestions.

The Suitability Assessment of Materials (SAM) for assessing the educational material’s suitability for use with the target audience was also used. It consists of a checklist divided into six domains: content; appropriate language for the population; graphic illustrations, lists, tables and graphs; layout and typography; stimulation for learning and motivation; and cultural appropriateness. The following scores were considered: 2 points – superior; 1 point – suitable; 0 point – unsuitable^(15-21)^.

All collection instruments hosted on Google Forms required a mandatory response to the items, except for the spaces intended for suggestions for changes in the educational material and some of the information related to education and profession, such as employment in more than one institution and graduate studies. This information was left open to better adapt to the characteristics of each professional and their view on the need for adjustments or not in certain aspects of the information booklet.

The entire process, from construction to validity and correction of the booklet, took place from November 2019 to February 2021.

### Data Analysis and Treatment

Data were obtained through Google Forms, which provides search results in ready-made tables in Excel. The data for calculating the CVC and the binomial test were then organized, coded, processed and analyzed using the Statistical Package for the Social Sciences (SPSS), version 20.0. To analyze the information booklet’s content validity, the Content Validity Coefficient (CVC) was calculated for each page of the booklet (_c_CVC) and for the material as a whole (_t_CVC), and an item whose agreement between content judges presented CVC > 0.80, and between technical judges, CVC > 0.70 was considered valid. This variation in the CVC value is predicted by the author in situations where judges have different backgrounds^(22)^, as is the case of the technical judges in this study. Moreover, the binomial test was calculated for each page of the booklet and p-value, where p > 0.05 confirms that the proportion of judges who considered the item relevant is equal to or greater than 80%, confirming the prepared educational material’s suitability and that the calculated CVC is valid.

The data obtained by applying the SAM questionnaire were organized and coded in the Excel program, with a percentage analysis of the scores achieved as follows: higher (70% to 100% of the scores); suitable (40 to 69%); and unsuitable (0 to 39%)^(15)^.

Judges’ suggestions were initially extracted from Excel and organized according to the type of judges (content or technical) and booklet pages. Suggestions were assessed by the researchers, quantified and categorized into content changes related to asthma, with adaptation of scientific terms and spelling, addition of information and changes to illustrations, layout, typography and diagramming.

After booklet validation and assessment by content and technical judges, their suggestions were analyzed, and a new contact was made with the professional responsible for the booklet illustration and diagramming so that they could carry out material modifications and adaptation, according to judges’ recommendations, especially in the pages that had a lower than expected result in the CVC and in SAM scores. Thus, the final version of the information booklet was obtained.

### Ethical Aspects

This research was approved by the Research Ethics Committee, under Opinion 3,845,712/2020. Data collection was only started after the approval and signing of the Informed Consent Form by content and technical judges.

The complete set of data is available at the *Universidade Federal do Ceará* repository. The information booklet in its final version, registered at the Brazilian Book Chamber, with IBSN and catalog file, is available for viewing, downloading and printing in supplementary files, hosted on an online platform or through direct request to the main author.

## RESULTS

**Figure 1:**
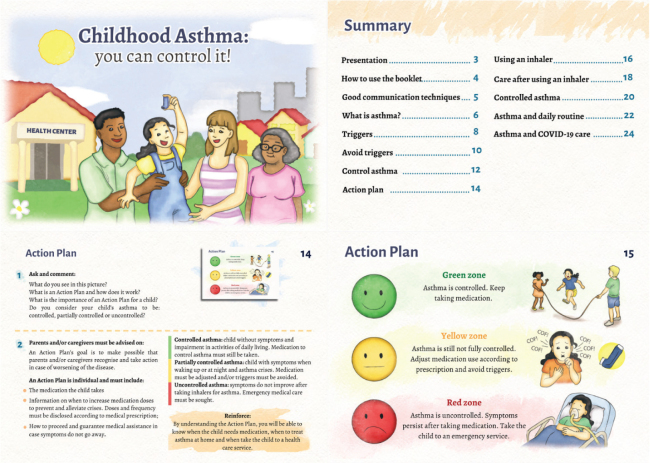
Pages from the booklet “Childhood asthma: you can control it!”. Fortaleza, CE, Brazil, 2021.

Source: own authorship. Illustration and diagramming by Joanna de Freitas Rocha.

The information booklet, entitled “Childhood asthma: you can control it!”, had its content organized and divided into four main themes: I – Introductory information for health professionals; II - Concept of asthma, symptoms and triggers of the disease; III - Asthma control and management; and IV – Asthma and COVID-19. The subjects addressed were divided into topics, as shown in the summary of the booklet (Figure 1).

In the booklet preparation, the main characters were presented on the cover, and the illustrations, watercolor style, portrayed an approximation with the target audience’s cultural reality. The information booklet totaled 28 pages, of which two are for the front and back cover, 20 for content about asthma, and six for pre-textual elements. Some are represented in Figure 1.

Content validity and appearance of the first version of the information booklet were carried out with 25 content judges (teaching and clinical nurses) and three technical judges (from the area of communication and design) from the South, Midwest and Northeast regions of the country. The judges’ average age was 39.9 + 9.73 years. Content judges had an average of 8.1 years of experience with asthma and 13.9 years with child health. Of the 25 judges, 24 (96%) had a master’s degree and 18 (72%) had a doctoral degree. Technical judges had an average of 6.6 years of experience with information booklets and 17.3 years of experience with printed materials. Of the three technical judges, two (66.6%) had completed specialization and master’s degrees, and one (33.3%) was a PhD. All judges met the previously mentioned Jasper’s selection criteria.

The _c_CVC was calculated for each page of the booklet, considering clarity of language, practical pertinence and theoretical relevance, represented in Table 1.

**Table 1:** Distribution of the _c_CVC for each page of the information booklet according to content judges’ (n = 25) and technical judges’ (n = 3) assessment. Fortaleza, CE, Brazil, 2020.

Pages/subjects	Language clarity				Practical pertinence				Theoretical relevance			
	CJ*	P^†^	TJ*	P^†^	CJ*	P^†^	TJ*	P^†^	CJ*	P^†^	TJ*	P^†^
Cover	0.90	0.97	0.83	1	0.94	0.97	0.76	0.48	0.93	0.99	0.76	0.48
Presentation	0.90	0.97	0.56	0.48	0.93	0.99	0.83	1	0.94	1	0.76	0.48
Using the booklet	0.96	1	0.76	1	0.98	1	0.83	1	0.98	1	0.83	1
Communication techniques	0.97	1	0.70	0.48	0.97	1	0.90	1	0.97	1	0.90	1
FR1 – What is asthma?	0.90	0.76	0.70	0.48	0.94	0.99	0.83	1	0.96	1	0.83	1
Fi1 – What is asthma?	0.93	0.97	0.90	1	0.96	0.99	0.90	1	0.96	0.99	0.90	1
FR2 – Trigger	0.95	0.97	0.76	0.48	0.97	0.99	0.90	1	0.97	0.99	0.90	1
Fi2 – Trigger	0.94	0.97	0.90	1	0.98	0.99	0.90	1	0.96	0.99	0.90	1
FR3 – Avoid triggers	0.96	1	0.76	0.48	0.98	1	0.90	1	0.98	1	0.90	1
Fi3 – Avoid triggers	0.98	1	0.76	0.48	0.98	1	0.83	0.48	0.98	1	0.83	0.48
FR4 – Asthma control	0.94	0.97	0.76	0.48	0.95	0.99	0.90	1	0.95	0.99	0.90	1
Fi4 – Asthma control	0.95	0.99	0.83	1	0.95	0.99	0.83	1	0.95	0.97	0.90	1
FR5 – Action plan	0.96	0.99	0.76	0.48	0.97	0.99	0.90	1	0.98	0.99	0.90	1
Fi5 – Action plan	0.98	0.99	0.90	1	0.98	0.99	0.83	1	0.98	0.99	0.90	1
FR6 – Using an inhaler	0.95	1	0.70	0.10	0.98	1	0.83	0.48	0.98	1	0.83	0.48
Fi6 – Using an inhaler	0.98	1	0.76	0.48	0.99	1	0.83	0.48	0.98	1	0.83	0.48
FR7 – Care after using an inhaler an inhaler	0.95	1	0.76	0.48	0.98	1	0.90	1	0.98	1	0.90	1
Fi7 – Care after using an inhaler an inhaler	0.94	0.99	0.83	0.48	0.98	1	0.83	0.48	0.98	1	0.83	0.48
FR8 – Controlled asthma	0.96	0.99	0.70	0.48	0.97	1	0.90	1	0.97	1	0.90	1
Fi8 – Controlled asthma	0.96	1	0.83	1	0.96	0.99	0.90	1	0.96	0.99	0.90	1
FR9 – Asthma and daily routine	0.98	1	0.76	1	0.98	1	0.90	1	0.98	1	0.90	1
Fi9 – Asthma and daily routine	0.98	1	0.70	0.10	0.98	1	0.83	0.48	0.98	1	0.83	0.48
FR10 – Asthma and COVID-19 care	0.97	1	0.76	0.48	0.97	1	0.90	1	0.98	1	0.90	1
Fi10 – Asthma and COVID-19 care	0.98	1	0.83	1	0.98	1	0.90	1	0.98	1	0.90	1
**TOTAL**	**0.95**		**0.77**		**0.97**		**0.86**		**0.97**		**0.87**	

Source: own authorship.

TJ – technical judges; CJ – content judges; *CVC – Content Validity Coefficient; P^†^ – binomial test.

Most booklet pages obtained a _c_CVC greater than 0.80 in the assessment carried out by content judges and 0.70 in the assessment of technical judges. However, the “presentation” page reached a value of less than 0.70 in relation to clarity of language, with changes being made to the text format, according to technical judges’ recommendations.

It is important to emphasize that the error calculation (PEi) to discount possible biases of judges was performed for each item, resulting in a value < 0.0001 for content judges and 0.037 for technical judges. This value was subtracted from the _i_CVC (initial CVC), and resulted in the values of _c_CVC (final CVC) shown in Table 1. The total CVC of the information booklet was 0.96, according to content judges, and 0.83, according to technical judges.

Furthermore, judges made suggestions for relevant changes in the educational material related to: changes in content related to asthma; suitability of scientific terms and spelling; addition of information; and changes to illustrations, layout, typography and diagramming. The main suggestions within each category are exemplified in Table 2. Only four content judges did not make any type of suggestion, question or comment throughout the booklet pages. Thus, there were 167 responses, from which 114 suggestions were extracted. All suggestions were analyzed by the researchers, and 69 were accepted.

**Table 2:** Categorization of suggestions (n = 69) made by content and technical judges for changes in the information booklet. Fortaleza, CE, Brazil, 2020.

Suggestions	n	%
**Change of content related to asthma**	**5**	**7.24**
Modification in the way and frequency of cleaning the spacer, changing the term puffer for inhaler, among others.
**Suitability of scientific terms and spelling**	**14**	**20.28**
Modification of scientific terms for words that are easier to understand, spelling and/or semantic changes, among others.
**Addition of information**	**26**	**37.68**
Summary page, concept of self-efficacy on the presentation page, concept of triggers for asthma exacerbation, incorporation of captions in some images, among others.
**Changing illustrations, layout, typography and diagramming**	**24**	**34.78**
Modification of typeface and increase in size, structuring the text in two columns, inclusion of a health center in the figure on the booklet cover.		

Source: own authorship.

Regarding educational material suitability assessment, according to SAM, the booklet was assessed as superior, having reached a percentage of agreement of 92.7% among content judges and 73.8% among technical judges. All assessed items described in the six domains obtained agreement, by content judges, above 70% (ranging from 78 to 98%), considered as superior.

However, technical judges rated 10 items (47.61%) as suitable (40% and 69%), and one item (4.76%) was classified as unsuitable in terms of layout and typography. The booklet’s degree of recommendation, measured by assigning a score from 0 to 10, obtained an average of 9,68 ± 0,54 by content judges and 9 ± 0,81 by technical judges.

## DISCUSSION

The information booklet presents a methodological foundation that confirms its validity and the possibility of being used by health professionals in strategies aimed at promoting self-efficacy of parents and/or caregivers of children with asthma. The validity process showed a technology with a high degree of agreement, and was classified by content and technical judges as an educational material with valid content and appearance.

The presentation page, although it did not reach a satisfactory assessment according to technical judges, with a value of less than 0.7, in language clarity, was not removed from the educational material, mainly due to its practical pertinence and theoretical relevance. Thus, the changes recommended by judges were made, in particular the insertion of the concept of self-efficacy.

Bandura’s Self-efficacy Theory is the information booklet’s theoretical framework, as it highlights that, as self-efficacy is developed, people intensify their efforts to achieve or even exceed the desired result^(23)^. Moreover, the use of a theory as the foundation of a health education action is essential, as it provides a plan of educational interventions that is more likely to succeed^(15)^. When considering the purpose of educational material as promoting self-efficacy of parents and caregivers in controlling and managing asthma, it is important that health professionals are aware of the theory’s concept.

Among the best assessed pages in the booklet, those on the factors that can trigger and/or exacerbate asthma symptoms – called “asthma triggers” -, action plan, care after using the inhaler with corticosteroids and COVID-19 prevention stand out.

Identification and prevention of triggers are secondary ways of preventing symptoms or exacerbations of asthma attacks. When caring for children with asthma, parents and/or caregivers must know how to recognize individual triggers. However, avoiding triggers can be difficult for families. It is important for health professionals to identify and list possible triggers, allergenic and non-allergenic, and ensure information on how to avoid them, according to the reality of each patient. However, in many cases, avoiding triggers is not a guarantee of successful disease control, requiring multifaceted outpatient follow-up^(24)^.

A written action plan helps with asthma management, especially about the signs and symptoms of an impending exacerbation and how to act in each case, including the use of prescribed medications^(25)^. The adequate use of an individualized action plan in childhood asthma reduces morbidity and mortality, visits to emergency services, days of hospitalization and school absenteeism^(25,26)^.

The importance of adding information on COVID-19 prevention in this booklet lies in the fact that, although asthma and the use of medications for its control are not risk factors for the disease, patients with asthma hospitalized for COVID-19, have a worse prognosis and higher mortality rate^(27)^.

In the assessment with SAM, the booklet was assessed as superior, with overall scores greater than 70% for both content and technical judges. In the SAM items whose booklet was assessed as suitable or unsuitable, it is important to highlight the recognition of some failures so that the researcher can assess the impact they may have on the understanding of the key information of the addressed content and take the necessary measures. In this regard, the material can have flaws corrected before being used with the target audience^(15)^.

Changes to the booklet were made based on judges’ suggestions and directed to the pages of the educational material that had lower assessments both in the aspects validated with CVC and in the items analyzed by SAM so that the content of the educational material is able to reach the target proposed goal.

Among the main suggestions, changes in the content related to asthma related to the way and frequency of spacer cleaning stand out. To make this change, spacer manuals from three different brands (Caretech, Agachamber and Incoterm) and the GINA manual were consulted^(1)^. When considering the divergent information present in the literature, the instructions for cleaning the spacer were modified, in order to meet the prerequisites considered most important, in the following stages: 1) clean weekly with detergent and rinse under running water; 2) immerse in a water solution added to two drops of detergent for 20 to 30 minutes, without rinsing; 3) air dry, without using paper or cloth.

Another suggestion for change was the replacement of the term “puffer” by “inhaler”. It is known that the use of scientific language must be limited and, in cases where a word is presented, the respective meaning must be explained using language appropriate for the audience^(16)^. Thus, inhaled medication was introduced in the text as an inhaler, explaining the purpose and the term “puffer”, by which it is more commonly known.

With the same justification regarding the limitation of scientific language, other terms, such as “cyanotic”, “nail bed” and “wheezing”, were replaced by terms that are easier to understand, as “purplish”, “nails” and “breathing sounds”, respectively.

Most of the suggestions corresponded to the addition of information, highlighting the inclusion of a summary page. The table of contents is important as it displays the booklet’s contents as a whole as an advertisement for the educational material. Users look in the table of contents to see if the material contains the information they are looking for^(28)^.

The concept of triggers was also added to the script sheet, which presents the main triggers for asthma exacerbation. This information is important, as asthma control and management largely depend on recognizing triggers. It should be noted that many patients or users of health services are not familiar with health care and disease treatment and that the lack of clarity in language can lead them to follow the guidelines inappropriately^(15)^.

It was also suggested to add subtitles to the following figures: actions to avoid triggers; using an inhaler; care after using an inhaler; controlled asthma; and daily activities performed by children with asthma. This suggestion was accepted, as it is understood that subtitles play a fundamental role in conveying the message that an image wants to spread. They must include the main information about the image and reinforce the message present in the material’s text^(16)^.

Changes related to the booklet’s illustrations, layout and typography and diagramming were also suggested and accepted. The main change made was the restructuring of the entire booklet text into two columns, previously presented in a single column. Columns are important to facilitate reading, as they bring rhythmic repetition and a basic pattern that give visual coherence to the material^(28)^.

Another alteration was the font of the text in the booklet, replacing the Times New Roman font, considered monotonous by some judges, for Alegreya, which is presented in both serif and sans serif versions. Priority was given to the use of sans-serif fonts for titles and subtitles and serif fonts for the body of texts, as the serif font promotes greater readability^(15)^.

The cover illustration was modified, with the addition of an image of a health center in the background of the main characters. This suggestion was accepted, as it recognizes the work of Primary Care health professionals in asthma management and control. In Brazil, Family Health Strategy professionals are responsible for monitoring all patients with asthma, including severe and difficult-to-control cases. Primary Care must ensure therapy compliance and use of the correct inhalation technique, with patients being referred to a reference center for joint follow-up only in case of difficulty in treating asthma^(29)^.

Thus, the final version of the educational material is the first information booklet on the subject aimed at parents/ caregivers of asthmatic children in Brazil and the first, in general, to bring spacer cleaning care and to address COVID-19. Another unprecedented aspect is that it was built based on the self-efficacy theory.

Despite the innovative character of the educational technology developed in the present study, the lack of validity with the target audience is pointed out as a limitation.

## CONCLUSION

The information booklet, called “Childhood asthma: you can control it!”, was prepared on a strong methodological basis and was considered valid in terms of content and appearance, reaching a total CVC of 0.96 for content judges and 0,83 for technical judges. The technology was assessed by judges as superior educational material using SAM, and reached a recommendation level of over 9 points. After validity and modifications suggested by judges, the booklet was considered suitable to promote self-efficacy of parents and/or caregivers of children with asthma in the disease control and management.

## Data Availability

Information booklet: www.bit.ly/albumasma.
